# H3K27me3 Loss in Central Nervous System Tumors: Diagnostic, Prognostic, and Therapeutic Implications

**DOI:** 10.3390/cancers16203451

**Published:** 2024-10-11

**Authors:** Giuseppe Angelico, Manuel Mazzucchelli, Giulio Attanasio, Giordana Tinnirello, Jessica Farina, Magda Zanelli, Andrea Palicelli, Alessandra Bisagni, Giuseppe Maria Vincenzo Barbagallo, Francesco Certo, Maurizio Zizzo, Nektarios Koufopoulos, Gaetano Magro, Rosario Caltabiano, Giuseppe Broggi

**Affiliations:** 1 Department of Medicine and Surgery, Kore University of Enna, 94100 Enna, Italy; giuseppe.angelico@unikore.it; 2 Department of Medical and Surgical Sciences and Advanced Technologies “G.F. Ingrassia”, Anatomic Pathology, University of Catania, 95123 Catania, Italy; manuel.mazzucchelli@virgilio.it (M.M.); attanasiogiulio@icloud.com (G.A.); giordanatinnirello@yahoo.it (G.T.); jessicafarina2693@gmail.com (J.F.); g.magro@unict.it (G.M.); rosario.caltabiano@unict.it (R.C.); 3 Pathology Unit, Azienda USL-IRCCS di Reggio Emilia, 42123 Reggio Emilia, Italy; magda.zanelli@ausl.re.it (M.Z.); andrea.palicelli@ausl.re.it (A.P.); alessandra.bisagni@ausl.re.it (A.B.); 4 Department of Neurological Surgery, Policlinico “G. Rodolico-S. Marco” University Hospital, 95121 Catania, Italy; gbarbagallo@unict.it (G.M.V.B.); cicciocerto@yahoo.it (F.C.); 5 Surgical Oncology Unit, Azienda USL-IRCCS di Reggio Emilia, 42122 Reggio Emilia, Italy; maurizio.zizzo@ausl.re.it; 6 Second Department of Pathology, Medical School, National and Kapodistrian University of Athens, Attikon University Hospital, 15772 Athens, Greece; nkoufo@med.uoa.gr

**Keywords:** H3K27me3 loss, central nervous system, tumor, diffuse midline glioma, immunohistochemistry

## Abstract

**Simple Summary:**

This article herein presented explores the role of the epigenetic marker H3K27me3 in tumors of the Central Nervous System, highlighting its importance for diagnosis, prognosis, and treatment. H3K27me3 involves the trimethylation of lysine 27 on the histone H3 protein, which is essential for regulating gene activity and maintaining chromatin structure. A reduction in H3K27me3 levels is commonly seen in CNS tumors such as diffuse midline gliomas and meningiomas and is associated with more aggressive tumor behavior and a worse prognosis. This article emphasizes the utility of H3K27me3 loss in tumor diagnosis, prognosis, and the advancement of targeted therapies.

**Abstract:**

Central nervous system (CNS) tumors represent a formidable clinical challenge due to their molecular complexity and varied prognostic outcomes. This review delves into the pivotal role of the epigenetic marker H3K27me3 in the development and treatment of CNS tumors. H3K27me3, specifically the trimethylation of lysine 27 on the histone H3 protein, plays a crucial role in regulating gene expression and maintaining chromatin architecture (e.g., in X-chromosome inactivation). Notably, a reduction in H3K27me3 levels, frequently tied to mutations in the H3 gene family such as H3F3A and HIST1H3B, is evident in diverse brain tumor variants, including the diffuse midline glioma characterized by the H3K27M mutation and certain pediatric high-grade gliomas. The loss of H3K27me3 has been linked to more aggressive behavior in meningiomas, with the trimethylation loss associated with significantly shorter recurrence-free survival (RFS) among grade 2 meningiomas, albeit not within grade 1 tumors. Pediatric posterior fossa ependymomas characterized by a lowered H3K27me3 and DNA hypomethylation exhibit poor prognosis, underscoring the prognostic significance of these epigenetic alterations in CNS tumors. Comprehending the role of H3K27me3 in CNS tumors is vital for advancing diagnostic tools and therapeutic interventions, with the goal of enhancing patient outcomes and quality of life. This review underscores the importance of ongoing investigations into H3K27me to refine and optimize management strategies for CNS tumors, paving the way for improved personalized medicine practices in oncology.

## 1. Introduction

Epigenetic mechanisms are central to the regulation of gene expression, mediating the response to environmental cues and cellular states without altering the DNA sequence itself. These mechanisms primarily involve modifications to DNA and histone proteins, which are key components of chromatin—the complex of DNA and proteins that form chromosomes within the nucleus of cells [1,2]. Histones are highly conserved proteins, which serve as spools around DNA and are tightly wound to form nucleosomes, each consisting of 147 base pairs of DNA wrapped around a histone octamer [3,4]. This octamer includes two copies each of the core histones H2A, H2B, H3, and H4. Linker histones, H1 and H5, bind to the DNA between nucleosomes, further compacting the chromatin and regulating access to the DNA [3,4,5]. The nucleosome is the fundamental unit of chromatin, organizing the genome into a higher-order structure that regulates gene accessibility and transcriptional activity [3,4,5]. Clinically, the H3K27me3 marker plays a pivotal role in the diagnosis, prognosis, and treatment of Central Nervous System (CNS) tumors. H3K27me3 involves the trimethylation of lysine 27 on the histone H3 protein, a modification essential for gene repression and maintaining chromatin structure. The loss of this marker is commonly associated with aggressive tumor behavior, particularly in CNS tumors such as diffuse midline gliomas and meningiomas. The reduction in H3K27me3 levels correlates with poor prognosis and shorter recurrence-free survival, especially in grade 2 meningiomas, although not in grade 1 tumors [3,4,5]. From a diagnostic standpoint, immunohistochemistry (IHC) is crucial in detecting H3K27me3 loss, providing insights into tumor classification and guiding treatment decisions. Targeted therapies focusing on histone modifications, such as those that restore H3K27me3 function, are emerging as promising approaches in managing these aggressive tumors [3,4,5]. Understanding the role of H3K27me3 in CNS tumors is essential for advancing diagnostic tools and therapeutic strategies, paving the way for improved patient outcomes and personalized medicine in oncology.

## 2. Post-Translational Modifications and Targeted Therapy

Post-translational modifications [PTMs] of histone proteins represent a vital mechanism for the dynamic regulation of gene expression, allowing for a nuanced control over the cell’s proteome in a cell-type-specific manner [6]. PTMs include a variety of covalent modifications such as methylation, phosphorylation, acetylation, ubiquitylation, SUMOylation, glycosylation, and ADP-ribosylation [6]. These modifications are primarily reversible, enabling the fine-tuning of chromatin’s physical properties, the recruitment of chromatin-binding proteins, and the overall regulation of gene expression [6].

Among these modifications, methylation and acetylation of lysine residues on histone tails are particularly well studied due to their significant impact on chromatin structure and function [6,7]. Acetylation is typically associated with transcriptional activation, as it leads to a loosening of the chromatin structure, making the DNA more accessible for transcription [6,7]. Methylation, on the other hand, can either activate or repress transcription, depending on whether lysine residue is modified and the extent of methylation [mono-, di-, or tri-methylation] [6,7,8].

The disruption of normal PTM pathways is linked to the development and progression of a wide range of human diseases, including cancer, heart failure, autoimmune disorders, and neurodegenerative diseases such as Parkinson’s, Alzheimer’s, and Huntington’s diseases [9,10].

Given the reversible nature of histone modifications, they have emerged as promising targets for therapeutic intervention, particularly in the field of oncology [11,12].

PTM-based therapies have been developed, targeting enzymes involved in the addition or removal of these modifications [11,12]. These therapeutic agents are categorized based on their target enzymes: histone methyltransferases [HMTs], histone demethylases [HDMs], histone acetyltransferases [HATs], and histone deacetylase inhibitors [HDACIs]. Notably, drugs such as the HMT inhibitor tazemetostat and HDACIs including vorinostat, romidepsin, belinostat, chidamide, and panobinostat have received marketing approval, offering new avenues for cancer treatment [13]. Other potential drugs targeting these modifications are in various stages of clinical development, underscoring the ongoing exploration of PTM-based interventions to mitigate the impact of cancer and metabolism-related diseases [13].

## 3. Histone H3 Family

The histone H3 family plays a pivotal role in chromatin structure and function, influencing gene regulation through various mechanisms [14]. This family includes several variants, such as H3.1, H3.2, and H3.3, which share a high degree of genetic similarity but differ slightly in their amino acid sequences [14]. These subtle variations are crucial as they determine how each variant interacts with chromatin, affecting DNA organization and accessibility for transcription [14].

H3.1 and H3.2 are considered canonical histone variants and are typically associated with the replication-dependent assembly of chromatin [15]. Their incorporation into chromatin primarily occurs during DNA replication in the S phase of the cell cycle [15]. These variants ensure that newly synthesized DNA is properly packaged into chromatin, maintaining its structure as cells divide [15].

H3.3, by contrast, is notable for its role in active gene expression. Unlike H3.1 and H3.2, H3.3 is incorporated into chromatin in a replication-independent manner [16,17]. This characteristic allows H3.3 to be integrated into chromatin at any stage of the cell cycle, not just during DNA replication [16,17]. This characteristic is particularly important for regulating gene expression, as H3.3 is frequently found in genomic regions that are actively being transcribed [16,17]. It is associated with enhancers, promoters, and other regulatory elements essential for transcriptional activity [16,17].

The incorporation of the H3.3 histone variant depends on specialized chaperone proteins that recognize its unique sequence features [18]. These chaperones transport H3.3 to sites of active transcription or repair, designating these regions for enhanced gene activity [18]. For instance, the chaperone Daxx, in cooperation with ATRX, facilitates chromatin assembly at telomeres independently of DNA replication [19]. The localization of H3.3 in these areas helps maintain a relaxed chromatin structure, making it more accessible to transcriptional machinery and thereby promoting gene expression [18,20].

Additionally, the selective integration of H3 variants into chromatin is crucial for attracting chromatin remodeling complexes and enzymes responsible for histone modifications [21]. These complexes play a key role in executing post-translational modifications on histones, which fine-tune the transcriptional activity of the associated genes [14,22]. For example, H3.3’s presence in gene bodies and regulatory domains can lead to modifications such as methylation and acetylation [14,22], which serve as critical markers for either activating or repressing gene expression [14,22].

## 4. H3K27me3: Pathophysiology

Methylation of lysine 27 on histone H3 (H3K27me3) is a vital epigenetic modification associated with gene repression [23], which plays a key role in regulating gene expression, crucial for developmental processes, cell differentiation, and the maintenance of cell identity [23,24]. The Polycomb Repressive Complex 2 (PRC2), containing either EZH2 (enhancer of zeste homolog 2) or EZH1 as catalytic components, is primarily responsible for adding the H3K27me3 mark [24,25]. This complex is central to the epigenetic landscape, facilitating the methylation that leads to gene silencing [23,24,25].

EZH2, in particular, is critical for the addition of methyl groups to H3K27, a process that compacts chromatin and suppresses transcription [26]. This modification is reversible, with demethylases such as KDM6A (UTX) and KDM6B (JMJD3) playing key roles in removing methyl groups from H3K27, thereby allowing the reactivation of previously silenced genes [27].

The H3K27me3 mark is a crucial indicator of transcriptionally inactive genes, particularly those involved in developmental pathways that must be silenced when not needed [28]. This gene silencing ensures the proper regulation of cell differentiation and the maintenance of cellular identity [28]. For instance, in stem cells, maintaining appropriate levels of H3K27me3 is essential for preserving pluripotency and ensuring timely differentiation into specific cell types [28]. This action is achieved by repressing differentiation-specific genes until the correct signals for differentiation are received [28].

Beyond development, H3K27me3 also plays a significant role in disease. Alterations in H3K27me3 levels, whether due to changes in EZH2 activity, PRC2 composition, or defects in demethylases, can lead to abnormal gene expression patterns [28,29]. Such disruptions are implicated in developmental disorders, where incorrect timing or levels of gene expression can lead to improper development [30,31]. In cancer, EZH2 overexpression has been identified in various tumor types, acting as an oncogene [32,33]. EZH2’s role in cancer involves the extensive silencing of tumor suppressor genes via H3K27me3, enabling uncontrolled cell growth and the evasion of cellular safeguards against cancer formation [32,33]. This dysregulation underscores the therapeutic potential of targeting H3K27me3 and its associated regulatory mechanisms [32,33]. Efforts to inhibit EZH2 or modulate H3K27me3 removal are being explored as strategies to reactivate tumor suppressor genes and slow cancer progression [32,33].

## 5. Immunohistochemistry Interpretation and Assessment by Pathologists

The interpretation of H3K27me3 staining through immunohistochemistry [IHC] is indispensable in diagnostic pathology, providing crucial insights into the epigenetic status of cells and tissues for disease diagnosis and classification [34,35]. H3K27me3, a histone modification linked to gene repression, exhibits distinct staining patterns in the nucleus under normal and disease-related conditions, detectable through IHC. The expression and distribution of H3K27me3 within the cell nucleus offer valuable diagnostic clues, underscoring the importance of considering context in interpretation [34,35].

Under normal conditions, H3K27me3 is consistently expressed across various cell types, displaying a diffuse nuclear staining pattern reflecting its role in regulating gene expression throughout the genome [34,35]. Deviations from this pattern can indicate disease-related changes, such as a complete or heterogeneous loss of H3K27me3 staining, forming a mosaic pattern, that can serve as a marker for certain diseases [35,36].

In the specific context of peripheral nerve sheath tumors, the H3K27me3 staining pattern is informative for distinguishing between malignant peripheral nerve sheath tumors [MPNSTs] and neurofibroma [36,37,38,39,40]. The loss of H3K27me3 staining is more frequently associated with MPNSTs, aiding in the differentiation from neurofibroma, which typically retains H3K27me3 expression [36,37,38,39,40]. This application highlights the value of histone modification patterns in accurately classifying tumors.

Furthermore, the eccentric intranuclear dot observed in H3K27me3 staining, particularly in normal tissues, signifies X-chromosome inactivation [41]. This normal epigenetic mechanism is crucial for balancing gene expression between XX females and XY males, resulting in the inactivation of one X chromosome [41,42]. The dot-like staining pattern serves as a distinctive marker for an inactivated X chromosome and is commonly employed in various diagnostic settings to assess the normality of cellular and tissue samples. Integrating H3K27me3 staining patterns, along with considering their context, enhances the accuracy of diagnostic evaluations in the field of pathology [41,42,43].

Conversely, increased H3K27me3 expression in cancers like oral squamous cell carcinoma suggests a worse prognosis, emphasizing the intricate role of this marker in cancer biology [25].

The wide applicability of H3K27me3 extends beyond MPNSTs and melanomas, making it a versatile biomarker in the pathology of various cancers. This characteristic underscores its significance in both the diagnosis and prognosis of cancer, offering pathologists a powerful tool for accurate classification and disease management [39,44]. Through the meticulous interpretation of H3K27me3 staining patterns, pathologists contribute to a deeper understanding of tumor behavior, facilitating the development of targeted therapeutic strategies. Pediatric-type diffuse gliomas frequently exhibit distinct mutations in the histone H3 gene, specifically H3F3A and HIST1H3B genes, leading to specific amino acid substitutions, such as H3K27M and H3G34R/V, and there is compelling evidence that these mutations correlate with the anatomic location of gliomas [45,46,47,48,49,50,51,52,53]. H3K27M mutations are predominantly found in midline gliomas, particularly in the brainstem, thalamus, and spinal cord [45,46,47,48,49,50,51,52,53]. These regions are associated with structures derived from the ventral neuroepithelium during embryonic development. The specific developmental pathways active in these midline regions may render them more susceptible to mutations that disrupt the epigenetic regulation controlled by H3K27; conversely, H3G34R/V mutations are frequently seen in cerebral hemispheric gliomas, particularly in younger patients [45,46,47,48,49,50,51,52,53]. The hemispheric location suggests that this mutation may arise in neural progenitor cells or differentiated neurons that populate these cortical areas. The distinct genetic environment and cellular context in these areas may be more prone to the epigenetic deregulation caused by H3G34 mutations [45,46,47,48,49,50,51,52,53].

Table 1 summarizes the main applications of H3K27me3 loss in CNS tumors.

## 6. Diffuse Midline Glioma, H3 K27-Altered

Diffuse midline glioma [DMG] is a diffuse glioma subtype arising from the brainstem (midbrain, pons and medulla), thalamus, spinal cord, pineal region, cerebellum, hypothalamus, or third ventricle [45,46]. It arises predominantly in children aged from 5 to 10 years of age, without gender predilection, and has a poor prognosis [45,46]. It can also occur in adolescents and adults arising predominantly from the thalamus (unilaterally) and spinal cord [45,46]. Diffuse intrinsic pontine glioma [DIPG] represents 10–15% of all pediatric brain tumors and 75% of all brainstem tumors, although thalamic DMGs are rarer [45,46].

Symptoms related to midline glioma depend on the tumor location, but usually, most patients present a short medical history (<2 months) with double vision, weakness, and ataxia on one or both sides of the body.

Magnetic resonance imaging (MRI) [45,46] is the gold standard imaging technique to diagnose DMG, especially when located in the pontine area. Typical findings are T1 and T2 hyperintense asymmetrical lesions with high perfusion and encasement of basilar artery. An exophytic component and midbrain, cerebellar peduncles or cerebellar hemispheres infiltration may be seen [45,46]. The integration of molecular and genomic features in histology plays a central role in the diagnosis panel, so much so that they lead many changes in the fifth edition of the WHO classification of CNS tumors, with DMGs classified as “Diffuse midline glioma, H3K27-altered”, corresponding to WHO grade IV, regardless of histologic characteristics [47].

While DMG diagnosis is usually based on clinical and radiological features, biopsies are fundamental to rule out other malignancies and to confirm the presence of H3K27 mutations, and therefore the loss of H3K27me3 [47,48]. Moreover, molecular studies can be performed to determine if other genetic alterations are co-occurring, since they may be responsible for a different outcome and can define the selection of clinical trials or targeted therapies [47,48].

**Figure 1:**
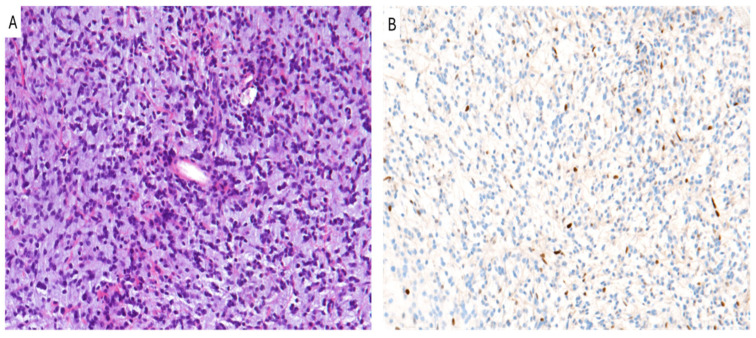
Diffuse midline glioma, H3 K27-altered. (**A**) An hypercellular diffuse glioma with astrocytic morphology and moderate/severe nuclear pleomorphism is seen. (**B**) Tumor cells show loss of nuclear staining for H3K27me3 (**A**,**B**): original magnifications 300x.

Tumor cells frequently exhibit astrocytic histologic features and small and monomorphic cells (Figure 1A), but they can occasionally show a more oligodendroglioma-like, giant cells and an undifferentiated or more epithelioid cytology [45,46,48]. Even though mitotic figures, necrosis, and microvascular proliferation may be seen, they are not required for diagnosis [45,46,48]. DMG shows an infiltrative growth pattern towards adjacent structures, with neurons entrapped within the neoplastic mass [45,46,48].

DMGs harbor somatic H3K27 mutations (H3K27M-mutant) that convert lysine to methionine at the 27th position from the N-terminus of the histone H3 tail, leading to the failure of a post-translationally modification that histone tails usually undergo, such as the loss of tri-methylation (H3K27me3) that, if normally expressed, represses transcription [45,46]. Hallmark genetic mutations of DMGs were found to be point mutations of the H3 gene, specifically H3F3A [H3.3] or HIST1H3B/C (H3.1) [47,49,50]. This global reduction in H3K27me3 results in an extensive transcriptional reprogramming of tumor cells, with the loss of gene silencing, prompting gliomagenesis by epigenetic regulation that increases cancer-related gene expression [47,49,50]. H3K27me3 is also fundamental to maintain chromatin structure, and its loss leads to genomic instability [47,49,50]. While those mutations were initially documented exclusively in DMGs, they were later reported in other brain tumors, such as ependymoma and non-diffuse pediatric low-grade astrocytoma and ganglioglioma [47,49,50].

Other mutations were described in DMGs, like an overexpression of the EZHIP and EGFR mutations. An EZHIP overexpression also results in the global reduction in H3K27-me3, as the EZHIP is the histone methyltransferase enhancer zest 2 (EZH2) inhibitory protein, normally deputed to permit the methylation of H3K27 [49]. Hence, a minority of DMGs can also be H3K27 wild-type (H3K27-WT) [49].

Immunohistochemistry is used to demonstrate the loss of H3K27me3 using a combination of H3K27M and H3K27-me3 antibodies that have a mutually exclusive immunoreactivity: nuclear staining of H3K27me3 is lost (Figure 1B), although H3K27M is strongly expressed [47,49,50]. This immunohistochemical pattern is typical of H3K27 mutated forms of DMGs [49,50]. However, in EZHIP overexpressed types of DMGs (H3K27-WT), immunohistochemistry shows a loss of immunoreactivity for both H3K27me3 and H3K27M [49,50]. In this case, an EZHIP immunohistochemical evaluation is fundamental, and it is found as overexpressed [49,50]. Therefore, the loss of trimethylation is mediated by either H3K27 mutations or an EZHIP overexpression [49,50].

Different parameters seem to have different prognostic impacts on DMGs. Apparently, DMGs do not behave the same in children and adults [45,46]. H3K27 mutations seem to have a very powerful impact as a prognostic parameter in children, although it is an uncertain marker of a dismal prognosis in adults [45,46]. In a recent study, Roberts et al. found a higher frequency of MAPK pathway alterations in long-term survival patients with DMG H3K27-altered compared to short-term survival patients. The most frequently mutated MAPK pathway genes were BRAF, NF1, and FGFR1 [51]. Moreover, the tumor location associated with the presence or lack of mutations seems to have a better understanding of the possible outcome [45,46]. Different subtypes of H3 mutations impact differently on overall survival (OS): the H3.1 mutation, found almost exclusively in the brainstem, shows better prognosis and response to treatment than H3.3 mutant gliomas, as the latter is present in 60–70% of diffuse intrinsic pontine glioma and along the midline, showing a median 11 months of OS [45,48].

Treatment options of DMGs are very limited. Chemotherapy usually uses temozolomide, carboplatin, and etoposide, but the outcome is still poor due to the intrinsic chemoresistance that DMG displays [45,46,47,48]. Radiotherapy is a staple in DMG treatment, mostly on those lesions that cannot be completely surgically resected [45,46,47,48]. Targeted therapy and immunotherapy use histone protein alterations to correct aberrant epigenetic modifications in order to restore a normal gene expression [45,46,47,48,49]. Inhibitors of EZH2 and DOT1L showed promising results in an early phase clinical trial [45,46,47,48]. However, genetic mutations and epigenetic alterations, such as DNA methylation and histone modifications, impacted negatively on response to treatment [49]. No significant difference was reported between the median survival rates of H3K27M and IDH/H3-WT gliomas, as the tumor location was the most impactful parameter in both classes of gliomas [45,48].

In contrast to diffuse gliomas with H3.3 p.G35R (G34R) or p.G35V (G34V) mutations that appear to be primarily hemispheric and to fulfill the diagnostic criteria for “diffuse hemispheric glioma, H3 G34-mutant”, it was reported that some diffuse gliomas with H3K27M mutations combined with the loss of H3K27me3 may rarely exhibit the lack of involvement of midline structures and do not fulfill the diagnostic criteria for DMG H3K27-altered [52]. According to the recommendations of the WHO classification, such cases should be reported as “diffuse hemispheric gliomas with H3K27M mutation not elsewhere classified (NEC)” [52]. Finally, although DMG H3K27-altered were initially classified as “pediatric-type gliomas”, it was demonstrated that they can also affect adolescents and adults, being usually monothalamic or spinal in these age groups [53].

## 7. Posterior Fossa Ependymoma

Posterior fossa ependymoma is a type of tumor affecting the Central Nervous System, and, according to the 2021 WHO classification, ependymal tumors are categorized based on histopathological, molecular features and their anatomical location [54,55]. These tumors are more common in the pediatric population, while spinal cord ependymomas are typically diagnosed in adults [55].

The new WHO classification of ependymal tumors lists two molecularly defined types of supratentorial ependymoma (with ZFTA or YAP1 fusion), two molecularly defined types of posterior fossa ependymoma, and a spinal tumor defined by the presence of MYCN amplification [54,55].

**Figure 2:**
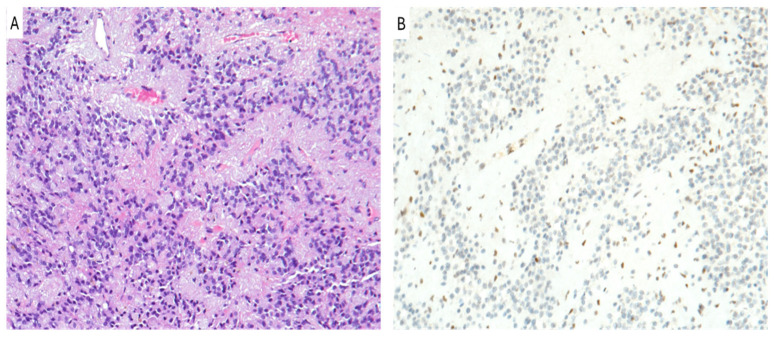
PFA ependymoma. (**A**) Histological examination showing a moderately cellular glioma composed of rounded cells forming perivascular pseudo-rosettes. (**B**) Neoplastic cells show the lack of immunoreactivity for H3K27me3 (**A**,**B**): original magnifications 300x.

Posterior fossa ependymomas can be assigned CNS WHO grade 2 or 3, based on brisk mitotic activity and microvascular proliferation, which are considered to have more prognostic impact than other histodisease-related features, such as nuclear pleomorphism or tumor necrosis (Figure 2A) [56]. However, efforts to risk-stratify cases based on histodisease-related grading criteria have yielded inconsistent results [57].

In the latest WHO classification, ependymomas of the posterior fossa are classified into two molecular subtypes: group A (PFEA) and group B (PFEB). Patients with PFEA are generally younger than those with PFEB [58]. An immunohistochemical analysis showed that the distinction between PFEA and PFEB can be reliably made based on immunoreactivity for H3K27me3, which has proven to be a high-quality and cost-effective test for differentiating the two groups [59,60]. This differentiation is prognostically significant, as PFEA exhibits a more aggressive behavior compared to PFEB [59,60]. In particular, a posterior fossa ependymoma may be classified as PFEA if a loss of H3K27me3 is identified either on immunohistochemistry (Figure 2B) or by DNA methylation profiling, whereas PFEB, spinal ependymomas, and posterior fossa subependymomas show a retained nuclear expression of H3K27me3 [60,61,62,63,64,65,66].

While DNA methylation profiling is the gold standard for molecular subgrouping, the loss of H3K27me3 by immunohistochemistry is now accepted as a surrogate marker for PFEA. The negative immunostaining for H3K27me3 (stained cells < 80% defined as negative, >80% defined as positive) was highly matched with PFEA, with specificity as high as 100%. Consistent with its association with a loss of expression in PFEA, H3K27me3 positivity was associated with better OS, representing an independent prognostic factor for OS [62,67].

Recent studies suggest that the loss of H3K27me3 in PFEA may be mediated by the overexpression of the EZH2 inhibitory protein (EZHIP) and by the involvement of the Elongin BC and Polycomb Repressive Complex 2-associated protein (EPOP), both of which play a role in the dysregulation of histone modifications [68,69]. Furthermore, CpG island (CpGi) hypermethylation and histone modifications, particularly H3K27 hypomethylation, are often found in these tumors, contributing to the poor prognosis observed in PFEA, as histological grading alone does not consistently predict outcomes in these cases [70]. A recent study conducted by Nambirajan et al. showed that the EZHIP diffuse and strong nuclear staining strongly associated with PFA group (*p* < 0.0001) and pediatric age group (*p* = 0.002) [58].

A subset of ependymomas, and, in particular, PFEA ependymomas, exhibits CpG island (CpGi) hypermethylation and histone modifications [in particular H3K27 hypomethylation], implicating epigenetic alterations in their pathogenesis, whereas recurrent somatic single-nucleotide variants (sSNVs) or copy number aberrations are rarely found in these tumors. For these tumors, a histological grade does not reliably predict prognosis. A genomic H3K27me3 distribution showed an inverse relationship with CpGi methylation, suggesting that CpGi hypermethylation drives low H3K27me3 in PFA ependymomas [64,70].

Recent studies showed that hypoxia maintains aberrant post-translational modifications of H3K27, demonstrating lower levels of H3K27 trimethylation in the hypoxic regions of PFEA ependymoma but not in supratentorial ependymoma controls (*p* = 0.0195) [70].

Determining H3K27me3 status by immunohistochemistry is a widely accepted method for molecular subgrouping of posterior fossa ependymomas. Despite the molecular distinctions between PFEA and PFEB, several studies emphasize that the extent of surgical resection remains a significant prognostic factor for posterior fossa ependymomas, regardless of molecular subgrouping [71,72]. This factor highlights the complex interplay between anatomical location, surgical feasibility, and molecular alterations in determining patient outcomes. Young age is an adverse prognostic factor in children with ependymomas. Treatment of these infants is challenging since beneficial therapeutic options are limited [73].

## 8. Oligodendroglioma, IDH-Mutant and 1p/19q-Codeleted

**Figure 3:**
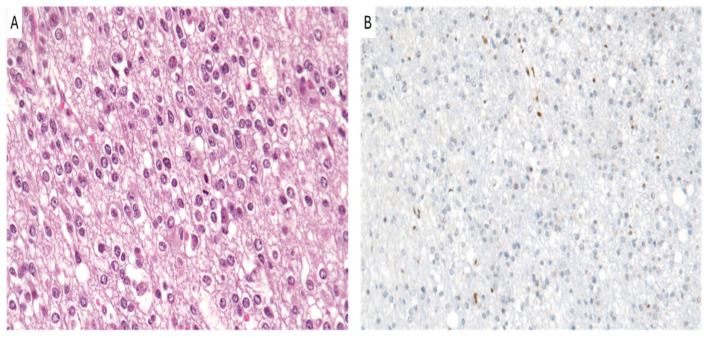
Oligodendroglioma, IDH-mutant and 1p/19q-codeleted. (**A**) A diffusely infiltrating glioma with rounded cells with perinuclear clear halo and brisk mitotic activity is seen. (**B**) The immunohistochemical loss of H3K27me3 is shown (**A**,**B**): original magnifications 300x.

The diagnosis of oligodendroglioma (Figure 3A) requires the presence of IDH1/2 mutation combined with 1p/19q codeletion assessed through a FISH analysis and/or molecular sequencing [74]. However, recent studies proposed surrogate immunohistochemical markers that could be used to identify 1p/19q codeletion without the need for more expensive tests. Specifically, based on their results assessing IDH mutations associated with ATRX and H3K27me3, immunostainings can help to determine the necessity of 1p/19q codeletion testing in IDH-mutant gliomas [75].

The potential use of H3K27me3 immunostaining for distinguishing between oligodendroglioma and astrocytoma was first described by Filipski et al. [75]. While the link between the loss of H3K27me3 immunoexpression (Figure 3B) and 1p/19q codeletion seemed to be confirmed in three separate studies [75,76,77], Pekmezci et al. discovered retained H3K27me3 in 7 out of 28 (25%) oligodendrogliomas and observed a co-occurrence of H3K27me3 loss along with retained or non-conclusive ATRX staining in 7 out of 74 (10%) IDH-mutant astrocytomas [78], emphasizing that H3K27me3 status alone cannot be used as an immunohistochemical surrogate for 1p/19q codeletion. It was proposed that the discordant results observed by Pekmezci et al. and Filipski et al. may be partially attributed to different concentrations of the same anti-H3K27me3 antibody (clone C36 B11) that they employed (1:50 vs. 1:200) [75,78]. Notably, Kitahama et al. demonstrated that a higher dilution of the antibody leads to increased specificity in predicting 1p/19q codeletion [79].

In a recent study by Ammendola et al. [80], the authors proposed a diagnostic algorithm based on the simultaneous testing of IDH mutations associated with ATRX and H3K27me3 immunohistochemistry [80]. Due to the observation that ATRX loss was restricted to the astrocytic phenotype, although the loss of H3K27me3 along with the retained ATRX was more consistent with the oligodendroglial phenotype, these authors suggested that 1p/19q codeletion testing could be reserved mainly to those IDH-mutant gliomas with retained ATRX and H3K27me3 immunohistochemical expressions [80].

Although multiple studies proposed the potential usefulness of H3K27me3 immunohistochemistry for predicting 1p/19q status, the results in this field are still controversial; therefore, the WHO Classification does not currently encourage the use of H3K27me3 as an immunohistochemical surrogate for 1p/19q codeletion in the diagnosis of oligodendrogliomas.

Moreover, H3K27me3 staining may also provide relevant prognostic information. Despite the limited number of studies on this topic, it has been suggested that retained immunostaining for H3K27me3 could be significantly associated with worse prognosis, independently from IDH mutational status and 1p/19q codeletion; on the other hand, H3K27me3 loss has been related to improved survival. These data are quite different from meningiomas and ependymomas, where H3K27me3 retention is associated with a better prognosis [80].

## 9. Meningioma

**Figure 4:**
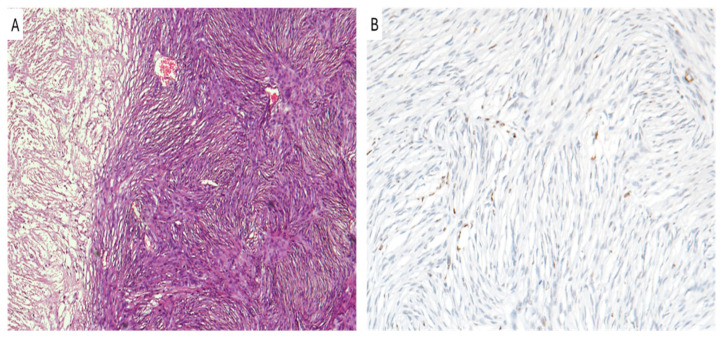
Meningioma. (**A**) WHO grade 3 anaplastic meningioma with pseudosarcomatous morphology and confluent necrotic areas is seen. (**B**) Neoplastic cells exhibit the loss of H3K27me3. (**A**,**B**): original magnifications 300x.

Meningiomas represent approximately 37% of all primary intracranial tumors and are the most common adult primary brain and spinal tumors [81]. They are classified as grade 1–3 tumors according to the CNS WHO grading system that is based on their histodisease-related and molecular features. In most cases, they are almost benign tumors (CNS WHO grade 1), which are different from the WHO grade 3 anaplastic meningiomas (Figure 4A) that are relatively rare, representing up to 4% of all meningiomas [81,82,83,84].

The management of meningiomas is complicated by different patterns of recurrence and their response to adjuvant radiotherapy [82].

In these tumors, the loss of H3K27me3 (Figure 4B) was recently identified as an indicator of potentially worse prognosis [83] and can be used as a marker for WHO grade 3 meningiomas [84]. The assessment of H3K27me3 staining by pathologists is crucial for tumor grading, and it has a significant role as a prognostic factor. In fact, in different studies, H3K27me3 loss was implicated in worse prognoses for patients affected by high grade meningiomas, and it also showed an increased risk of tumor recurrence, just after tumor chirurgical resection [85,86,87,88].

We must admit that, in each study that was reported about this correlation since now, there were some difficulties analyzing H3K27me3 loss by IHC, quantifying its impact, interpreting its clinical usefulness, and picking the right samples just to obtain reliable results [84,85,86,87,88].

Further studies need to be performed to enhance the correlation between H3K27me3 loss and grade 3 meningiomas in order to create better and efficient therapeutic strategies and to lower the incidence of recurrency after surgical resection.

## 10. Therapeutic Implications

Recent advances in post-translational modification (PTM)-based therapies primarily focused on targeting the enzymes responsible for histone modifications that are pivotal in regulating gene expression and contributing to the pathogenesis of various cancers, including CNS tumors [89,90,91,92]. One of the most promising approaches in this field involves targeting EZH2, a histone methyltransferase that catalyzes the trimethylation of lysine 27 on histone H3 (H3K27me3). EZH2 inhibitors, such as tazemetostat, have garnered significant attention due to their therapeutic potential [89,90,91]. Tazemetostat is already approved for treating epithelioid sarcoma and certain lymphomas and is currently under investigation for its efficacy in Central Nervous System (CNS) tumors, particularly those harboring alterations in H3K27me3 [89,90,91,92]. Early stage clinical trials are exploring its use in gliomas and other CNS malignancies where the loss of H3K27me3 is implicated in tumorigenesis. Some authors reported the potential use of tazemetostat in a subset of pediatric brain tumors with a high H3K27me3 expression, suggesting that EZH2-negative neoplastic cells might contribute to therapy resistance [93]. Additionally, Sharma et al. identified a dual EZH2/HSP90 inhibitor with significant inhibitory effects on cell growth in temozolomide-resistant GBM cell lines through in vitro studies [94]. Rahal et al. tested a chemical EZH2 inhibitor (GSK126) on DMG cell lines and explored metabolic therapeutic responses; they found that GSK126 induced sensitivity to cholesterol biosynthesis inhibitors, which could be further investigated as a potential therapeutic approach [95]. Beyond EZH2 inhibitors, novel agents are being developed to target non-histone PTMs, which also play critical roles in tumor progression, especially in CNS tumors [89,90,91,92]. Dordaprivone, a novel agent under investigation, modulates phosphorylation pathways that interact with epigenetic regulation. By addressing not only the loss of H3K27me3 but also other phosphorylation-related PTM mechanisms, dordaprivone opens new therapeutic avenues. Its ability to influence PTMs beyond histones offers a promising strategy to tackle the complexity of epigenetic alterations in aggressive CNS tumors [89,90,91,92]. Combination therapies are emerging as a robust approach in the treatment of H3K27me3-deficient CNS tumors. Preclinical models demonstrated that pairing H3K27-targeted therapies, such as EZH2 inhibitors, with histone deacetylase inhibitors (HDACIs) could be particularly effective. HDAC inhibitors like panobinostat and vorinostat block the deacetylation of histones, leading to the reactivation of tumor suppressor genes that had been silenced through epigenetic modifications [89,90,91,92]. The synergistic effects of combining HDACIs with H3K27-targeted therapies offer a multi-faceted approach to disrupting tumor growth by addressing multiple epigenetic pathways simultaneously, thus increasing the potential therapeutic efficacy in these aggressive tumors. As research progresses, a deeper understanding of the complex interactions between H3K27me3 and other PTMs is facilitating the development of more personalized cancer therapies. The future of CNS tumor treatment could see the rise of multi-targeted inhibitors that not only tackle the loss of H3K27me3 but also address other co-occurring PTM- related aberrations [89,90,91,92]. Such inhibitors could provide more tailored and precise treatment options, potentially improving outcomes for patients with CNS tumors that exhibit PTM-driven mechanisms of resistance and recurrence [89,90,91,92]. By integrating the latest advances in PTM-targeted therapies, there is significant potential to enhance survival rates and reduce the recurrence of these highly aggressive CNS tumors. With ongoing clinical trials and preclinical studies supporting the efficacy of both EZH2 inhibitors and combination therapies, the landscape of treatment for CNS tumors is poised for transformative change, offering new hope for patients facing these challenging cancers.

## 11. Conclusions

Numerous recent studies highlighted the loss of H3K27me3 in various CNS tumors, even in structures other than those of the midline, so it is currently well known that its detection is not restricted to DMGs, H3 K27-altered. In addition to the neoplasms covered in this review, H3K27me3 loss was reported to predict a significantly better prognosis in diffuse IDH-mutant hemispheric gliomas [80], although it appeared to have no prognostic impact in rosette-forming glioneuronal tumors [96]. Recently, it was also reported that H3K27me3 loss could be found in central neurocytomas and pituicytomas, in the absence of H3F3A mutations and EZHIP overexpression [97]. Finally, we would like to emphasize that the neuropathologists must be aware that a broad spectrum of CNS tumors with different prognoses may exhibit the immunohistochemical loss of H3K27me3 to avoid potential misdiagnoses, especially when dealing with small biopsies. In this regard, we propose to include H3K27me3 into the immunohistochemical panel when dealing with all the tumor entities discussed in the present review, in order to provide clinicians with proper prognostic and therapeutic information.

## Figures and Tables

**Table 1:** H3K27me3 loss in central nervous system tumors.

Tumor Type	H3K27me3 Status	Prognosis	Therapy
Diffuse midline glioma, H3 K27-altered	Loss of H3K27me3 (H3K27M)	Poor prognosis, short survival	EZH2/DOT1L inhibitors, Radiotherapy
Posterior Fossa Ependymoma	Loss of H3K27me3 in Group A (PFEA)	Poor prognosis in Group A (PFEA), better in Group B (PFEB)	Surgery, Radiotherapy, possible Chemotherapy
Oligodendroglioma, IDH-mutant and 1p19q-codeleted	Retained or loss of H3K27me3	Depends on 1p/19q co-deletion; worse with H3K27me3 loss	Chemotherapy, Molecular targeted Therapy
Meningioma (grade 2 and 3)	Loss of H3K27me3	Higher recurrence, worse prognosis	Epigenetic therapies, Radiotherapy

## Data Availability

No new data were generated in this article.
